# Protein Dielectrophoresis with Gradient Array of Conductive Electrodes Sheds New Light on Empirical Theory

**DOI:** 10.1021/acs.analchem.2c04708

**Published:** 2023-01-24

**Authors:** Siarhei Zavatski, Hanna Bandarenka, Olivier J. F. Martin

**Affiliations:** † Nanophotonics and Metrology Laboratory (NAM), Swiss Federal Institute of Technology Lausanne (EPFL), Lausanne1015, Switzerland; ‡ The Polytechnic School, Arizona State University, Mesa, Arizona85212, United States

## Abstract

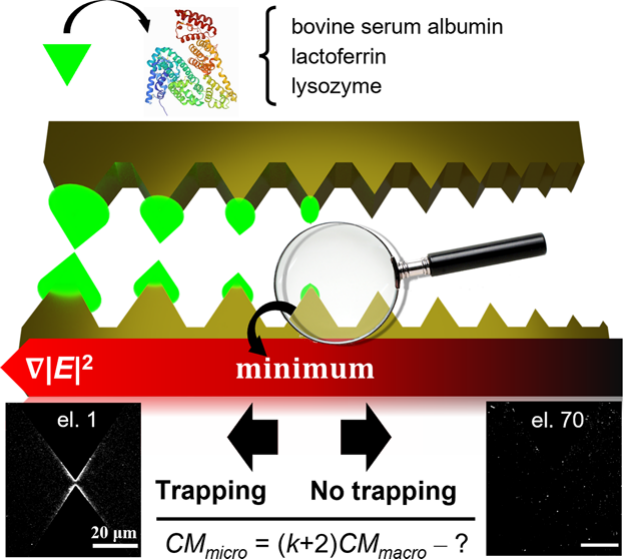

Dielectrophoresis (DEP) is a versatile tool for the precise microscale manipulation of a broad range of substances. To unleash the full potential of DEP for the manipulation of complex molecular-sized particulates such as proteins requires the development of appropriate theoretical models and their comprehensive experimental verification. Here, we construct an original DEP platform and test the Hölzel–Pethig empirical model for protein DEP. Three different proteins are studied: lysozyme, BSA, and lactoferrin. Their molecular Clausius–Mossotti function is obtained by detecting their trapping event via the measurement of the fluorescence intensity to identify the minimum electric field gradient required to overcome dispersive forces. We observe a significant discrepancy with published theoretical data and, after a very careful analysis to rule out experimental errors, conclude that more sophisticated theoretical models are required for the response of molecular entities in DEP fields. The developed experimental platform, which includes arrays of sawtooth metal electrode pairs with varying gaps and produces variations of the electric field gradient, provides a versatile tool that can broaden the utilization of DEP for molecular entities.

The spatial manipulation of proteins using an external force is of paramount interest for both fundamental studies and real-world applications,^1−5^ such as developing new types of biosensors for medicine,^6,7^ biology,^8,9^ food industry,^10,11^ ecology,^12,13^ to name a few. Dielectrophoresis (DEP), representing a motion of particulates induced by an inhomogeneous electric field (EF), is a solid candidate for such manipulations, and several experiments have demonstrated the separation, focusing, and immobilization of proteins using DEP.^14−20^ There is, however, still a lack of complete understanding of the mechanisms involved in this process, and no solid theoretical model exists. In contrast, colloidal nanoparticles and cell DEP have been explained in terms of a well-developed classical dielectric theory,^21^ which validity has been broadly confirmed experimentally.^22−25^

Recently, Hölzel and Pethig have discussed in detail the existing “classical” DEP theory limitations when applied to interpret the experimental manipulation of proteins.^26,27^ Briefly, in these series of studies, they stressed that, starting with the very first report on protein DEP,^28^ most experiments have been realized by applying electric field (EF) gradients several orders of magnitude lower than the minimum value predicted by the standard DEP theory to overcome dispersive forces associated with Brownian motion.^29^ The reason for this surprising disagreement is most probably connected with the fact that a protein carries net positive or negative charges, which constitute its permanent dipole moment and are responsible for the so-called dielectric dispersion.^30,31^ Hence, the total dipole moment of a protein in a DEP experiment should be the sum of the permanent and induced dipole moments, while the classical DEP theory deals only with the latter. It is important to emphasize that “the protein′s dipole moment” refers to that originating from the protein structure (polypeptide chain), the protein hydration sheath, and the water molecules electrostatically attracted by the protein. As a result, the actual polarizability of this dynamic molecular conglomerate becomes several orders of magnitude larger than the polarizability predicted by the standard macroscopic theory,^27^ and the EF gradient required for DEP is considerably reduced.

Since both dipole moments are likely to simultaneously exist upon exciting the protein in an inhomogeneous EF, the standard DEP theory thus must describe them jointly in some way, which is currently not the case. By considering this, Hölzel and Pethig have also made an attempt to reconciliate both polarization mechanisms by deriving a simple empirical relation, which involves the dielectric increment Δε—the bridge between microscale polarization processes in the protein–water mixture and its macroscale experimental determination—available from dielectric spectroscopy data, providing a practical model for analyzing protein DEP, at least until reliable ab initio calculations are available for all proteins of interest. Actually, there are some promising developments in this direction: Matyushov and colleagues have developed an analytical theory that includes the correlation terms between protein and water dipole moments and accounts for the close interaction between two dielectrics in a mixture, which is neglected in classical DEP theory.^32,33^ This analytical theory, coupled with the massive molecular dynamics simulations, represents a tremendous step toward a better understanding of protein DEP.

This excellent idea of linking micro- and macroscales for proteins using experimentally available data on both the DEP force and the dielectric increment Δε attracts a large interest for practical verification. Unfortunately, among nearly 22 different proteins studied by means of DEP as reviewed elsewhere,^26^ the required dielectric spectroscopy data exist only for three of them, namely, bovine serum albumin (BSA),^34^ concanavalin,^35^ and ribonuclease.^35,36^ Yet, the calculations using the proposed model for these three proteins are very insightful;^26,27^ the estimated DEP forces become closer to those observed experimentally.^17,28^ It is therefore of practical importance to expand the pool of proteins that can be described correctly with this simple model.

We thus study here the DEP responses for three proteins, namely, lysozyme, lactoferrin, and BSA. The choice of this set is dictated by the varying availability of dielectric spectroscopy and DEP data: BSA has been completely studied,^14,17,34^ lysozyme has been partly studied (only one recent experimental DEP study),^20,35^ while no DEP or dielectric spectroscopy data are available for lactoferrin. This way, we are able to both test the reliability of our experimental approach for determining the different DEP parameters and generate experimental DEP data for an additional protein.

The DEP device designed for this study provides a spatial variation of the EF gradient magnitude by varying the gap distance *g* between two opposing sawtooth electrodes (Figure 1a). In contrast to the vast majority of electrode patterns developed to date, which are based on insulator (iDEP) or conductive (eDEP) electrodes separated by a fixed *g*,^14,17,37−41^ the significant advantage of our platform is an additional degree of freedom to control the EF gradient magnitude. This intrinsic EF gradient control by the electrode geometry is especially suitable to investigate complicated species mixtures because the required EF gradient magnitude to overcome the dispersive forces and thus the correct electrode gap and applied voltage may not be known beforehand. This knowledge is not a prerequisite when utilizing this device with varying *g* since many different EF gradients and therefore DEP forces can be generated at once. This saves experimental time since one does not need to search for a suitable voltage. Moreover, by an accurate choice of the *g* increments, this voltage may be considerably reduced, which helps eliminate damages and/or destructive electrothermal effects.^42−45^ Finally, a single gradient DEP platform may contain many different electrodes with steep or gradual *g* variations, thus increasing device application flexibility. We should, however, note that the DEP device presented here is not suitable for the negative DEP of proteins.

**Figure 1:**
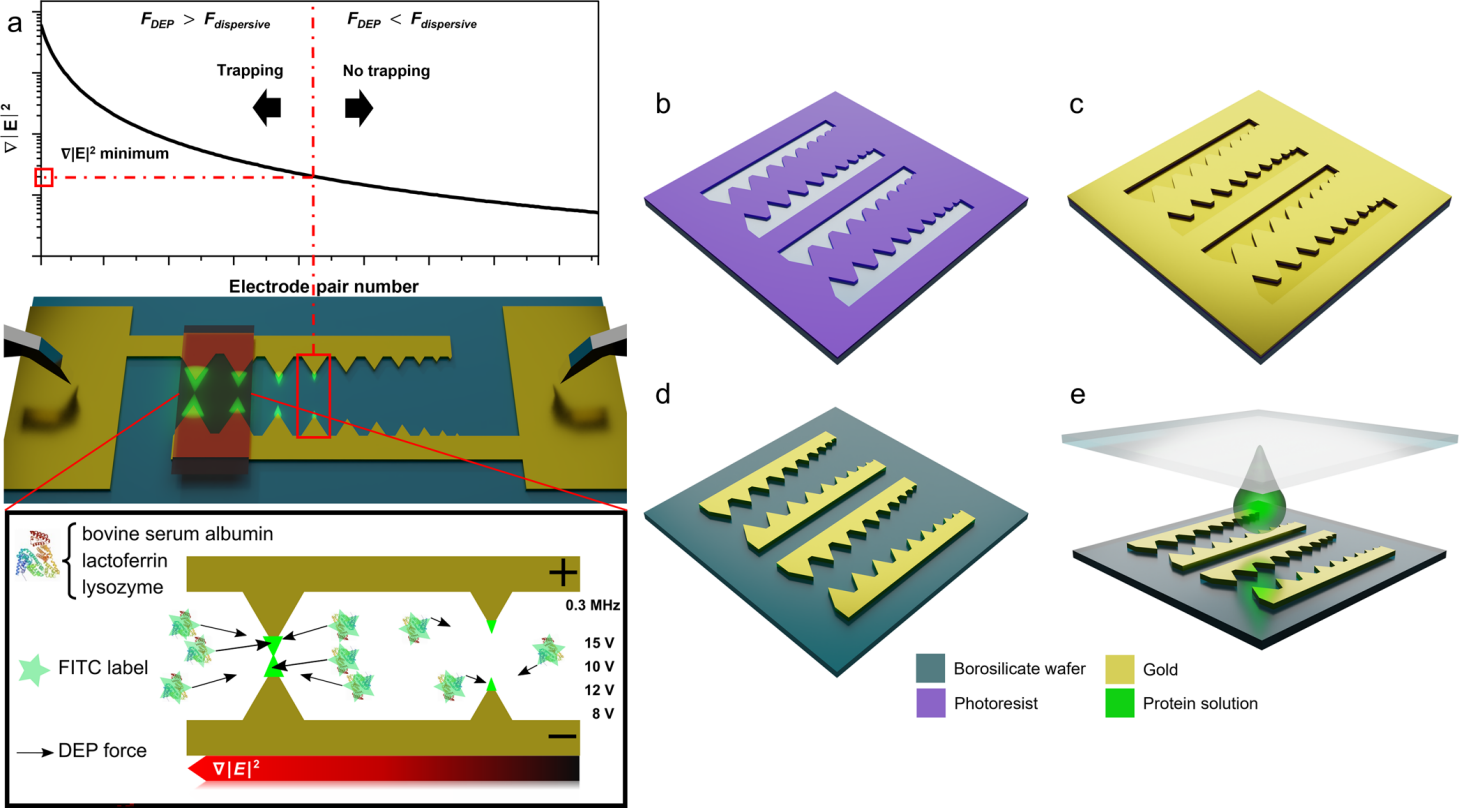
(a) Working principle of the dielectrophoretic device utilized to determine the molecular CM_micro_ for lysozyme, BSA, and lactoferrin. Schematic representation of the fabrication process for the device utilized in this work: (b) photoresist coating and exposure, (c) Ti–Au layer deposition by electron beam evaporation, (d) the resulting structure after the lift-off step, and (e) chamber preparation for DEP experiments.

Despite the apparent advantages of utilizing varying *g* DEP devices, to the best of our knowledge, only one very recent study, which has appeared in the literature at the time of the present manuscript preparation, has demonstrated a similar DEP platform design,^20^ although operated under very high DC fields, while our device is eDEP and excited with a moderate AC electric signal.

We demonstrate a discrepancy of 3.5 times for lysozyme and 2 orders of magnitude for BSA between our experimental data and the Hölzel–Pethig empirical model. The results are obtained by a combination of quantitative fluorescence microscopy, accurate three-dimensional (3D) finite-element method (FEM) numerical simulation, and state-of-the-art scanning electron microscopy (SEM) augmented by focused ion beam (FIB) SEM characterization. All of the results are also carefully analyzed for various possible sources of experimental errors, showing their minor effect.

## DEP Device Working Principles

The following modification of “classical” DEP theory has been proposed for more accurate protein DEP behavior predictions but still requires experimental verification^26^
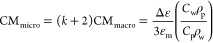
1where *C*_w_ and *C*_p_ are the molar concentrations of pure water (55.5 M) and protein, respectively; ρ_w_ and ρ_p_ are the mass densities of pure water and protein, respectively; and ε_m_ is the dielectric constant of the solvent (ε_m_ = 78 in our study, which is the dielectric constant of water). Values for Δε have been taken from the corresponding dielectric spectroscopy measurements (see Table 1 reported by Hölzel and Pethig).^26,27^ Values for ρ_p_ have been derived using their molecular-weight dependency.^46^

**Table 1:** Comparison of Different ∇|*E*_0_|^2^ and CM_micro_ Values for Three Proteins Studied in This Work^a^

protein	minimum ∇|*E*_0_|^2^ required to overcome the dispersive force as predicted by standard macroscopic DEP theory, V^2^/m^3^	experimental minimum ∇|*E*_0_|^2^ required to overcome dispersive force, V^2^/m^3^	experimental CM_micro_ calculated by eq 3	experimental CM_micro_ calculated by using *V*_Fr_	reported CM_micro_
lysozyme	7.29 (2.98) x 10^22^	[1.84 ± 0.24] x 10^20^	402 ± 50	94 ± 12	1′390
			(166 ± 23)		
BSA	3.18 (1.60) x 10^21^	[6.52 ± 0.84] x 10^20^	5 ± 0.65	0.36 ± 0.05	1′110
			(2 ± 0.27)		
lactoferrin	1.87 (0.20) x 10^21^	[6.44 ± 0.98] x 10^20^	3 ± 0.50	N/A	N/A
			(0.32 ± 0.5)		

a Reported CM_micro_ values in the last column are those derived elsewhere.^26,27^

We therefore take this challenge up by utilizing a novel DEP device depicted in Figure 1. Its working principle is as follows. When the protein is subjected to spatially varying EF gradients ∇*E*_0_^2^, it experiences different DEP forces, which are directed toward electrodes in our study and defined as
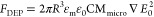
2where *R* is the hydrodynamic radius of the protein (according to the literature: 1.6 nm for lysozyme,^33^ 3.5 nm for BSA,^47^ and 4 nm for lactoferrin.^48^ This is, however, slightly different from our dynamic light scattering measurements reported in the Supporting Information Figure S1) and ε_0_ is the vacuum permittivity.

The experimental conditions are chosen such that other confounding forces (e.g., electrothermal and electrophoretic forces) and protein aggregation in the sample can be neglected. Thus, proteins are trapped by each successive electrode pair, up to the one that provides insufficient EF gradient magnitude to generate large enough DEP force to overcome dispersive forces associated with the Brownian motion. The trapping event is observed in terms of fluorescence intensity. Progressing along the successive electrode pairs with increasing gap distances shows a sudden fluorescence intensity drop: from that position onward, no protein trapping and therefore no fluorescence is observed for electrode pairs with larger gaps. The EF gradient magnitude estimated by numerical calculations for the last electrode pair with observable fluorescence (threshold electrode) gives the minimum EF gradient required to trap the chosen protein. Applying the empirical model from eq 1 and taking the maximum dispersive force acting on proteins equal to −0.5*KT R*^–1^, defined by R. Pethig in his book on p. 352,^29^ where *K* is the Boltzmann constant, we estimate the molecular CM_micro_ function using
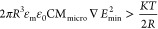
3

Strictly speaking, eq 3 provides a lower bound for CM_micro_; however, it is reasonable to assume equality between both terms at the trapping threshold. The experimentally obtained molecular CM_micro_ functions for each protein are then compared with reported values.^26,27^

## Experimental Section

### Chemicals

Fluorescein isothiocyanate molecules (FITC, excitation 490 nm, emission 525 nm), carbonate–bicarbonate capsules, 4-(2-hydroxyethyl)-1-piperazineethanesulfonic acid powder (HEPES, ≥99.5%), sodium hydroxide powder, dimethylsulfoxide (DMSO), lysozyme from chicken egg white (14.3 kDa, ≥90%), BSA (66 kDa, ≥98%), lactoferrin from bovine colostrum (85 kDa, ≥85%), and dialysis tubes (Pur-A-Lyzer tubes, molecular-weight cutoff 3.5 kDa) were purchased from Sigma-Aldrich. Syringe filters (0.1 μm) with alumina membrane were obtained from Whatman Anotop. Imaging spacers were acquired from Grace Bio-Labs SecureSeal.

### Dielectrophoretic Device Fabrication

Standard microelectronics techniques were employed for the dielectrophoretic device fabrication.^49,50^ First, a 100 mm borosilicate wafer (D263T/DS) was cleaned in piranha solution (UFT piranha wet bench) followed by treatment in high-frequency oxygen plasma (Tepla GiGabatch) at 600 W for 3 min with a 400 sccm O_2_ flow. Next, a negative photoresist AZ nLOF 2020 was spin-coated at 6000 rpm for 45 s and baked on a hot plate at 105 °C for 60 s, producing a 1.4 μm thick photoresist layer (Figure 1b). The microelectrode pattern was exposed using an i-line photoresist laser writer (Heidelberg VPG 200). Because AZ nLOF 2020 is an image reversal photoresist, a postexposure bake step was performed at 105 °C for 75 s to complete the photoreaction. The photoresist development was completed by dipping the wafer in an AZ 762 MIF developer for 75 s, followed by vigorous washing with deionized water and drying in a nitrogen flow. Prior to metal evaporation, an additional descum step in O_2_ plasma of 200 W applied for 10 s was performed to remove organic debris and increase the metal adhesion to the glass wafer. A layer consisting of 5 nm of Ti and 100 nm of Au was evaporated onto the patterned wafer by a Leybold Optics LAB600H electron beam evaporator (Figure 1c). A subsequent lift-off step was carried out by sonication of the coated wafer in an SVG 14 remover for 10 min at 75 °C, followed by a thorough cleaning with IPA and drying with N_2_ (Figure 1d). Finally, freshly prepared wafers were diced to produce 31 × 25 mm^2^ dielectrophoretic chips and were kept in a nitrogen chamber prior to experiments.

### Numerical Simulations

The electromagnetic finite-element simulations for the dielectrophoretic device were performed using the AC/DC module of COMSOL Multiphysics 5.6. The calculation procedure was based on the simulation of the potential ϕ distribution by solving the Laplace equation: ∇^2^ϕ = 0. The electric field thus was obtained as *E* = −∇ϕ and ∇*E* = ∇(−∇ϕ) for its gradient. Simulations were conducted for each *N*_e_ electrode pair by performing a parametric sweep through the gap size *g*. One of the electrodes from the pair was grounded, while the boundary condition for the second was ϕ = *V*_pp_, where *V*_pp_ = 8, 10, 12, and 15 V at an applied frequency of 0.3 MHz. The field was simulated in a water background, assuming a dielectric permittivity ε_m_ = 78 and an electrical conductivity σ_m_ of 167.5 μS/cm. Gold with σ_gold_ = 456 kS/cm and ε_gold_ = 6.9 was used for the electrode material. The mesh size used for the discretization was 5 nm (see the Supporting Information, Figure S3).

### Protein Labeling

All proteins utilized in this work were labeled with FITC according to the following procedure. First, one carbonate–bicarbonate capsule was dissolved in deionized water (50 mL) producing buffer solution with pH 9.5. Next, HEPES (2.38 g) was dissolved in deionized water (90 mL) and mixed with NaOH (2.5 mL, 2 M) to adjust the pH. The volume of the buffer was increased up to 100 mL and then dissolved with deionized water to obtain HEPES solution (5 mM) with a pH of 7.4 and a conductivity of 167.5 μS/cm. Next, FITC (1 mg) was dissolved in dry DMSO (1 mL) immediately before usage to avoid its degradation with time. A solution of lysozyme (7 mg/mL), BSA (7 mg/mL), and lactoferrin (7 mg/mL) in carbonate–bicarbonate buffer was mixed with FITC (276, 58, and 31 μL, respectively) in DMSO. The mixtures were vigorously stirred for 1 h at room temperature in the dark. After the reaction occurred, the mixtures were filtered using 0.1 μm syringe filters and dialyzed against HEPES buffer (1 L, 5 mM) for 14 h to remove the excess of unbound dye molecules. The dialyzed solution was again filtered and adjusted to the final concentration for each protein (500 ng/mL).

### Characterization

The reproducibility and morphology of the fabricated dielectrophoretic device were characterized by field-emission SEM (Zeiss MERLIN) and FIB SEM (FEI Nova 600 NanoLab) managed at 3 and 5 kV, respectively. An ultraviolet–visible–near-infrared (UV–vis–NIR) spectrophotometer (Shimadzu UV-2600) was utilized to determine the degree of labeling for the proteins.

### DEP Experiments and Postprocessing

For the protein DEP experiments, a drop of freshly labeled protein solution (37.7 μL) was placed on top of the dielectrophoretic device, covered with an imaging spacer (Grace Bio-Labs SecureSeal), and then sealed with a microscope coverslip (Figure 1e). The device was connected to the function generator (GW Instek AFG-2125) using macroelectrodes by making a mechanical contact with the contact pads. All of the experiments were conducted by the application of a sinusoidal voltage waveform with a frequency of 0.3 MHz and a peak-to-peak voltage of 8, 10, 12, and 15 V for 10 min. Subsequently, confocal fluorescent images for different electrode pairs were acquired by spinning disc microscope (Visitron SD CSU W1) and saved for further quantitative analysis by image processing software (ImageJ).

## Results and Discussion

### Device Design and Fabrication

**Figure 2:**
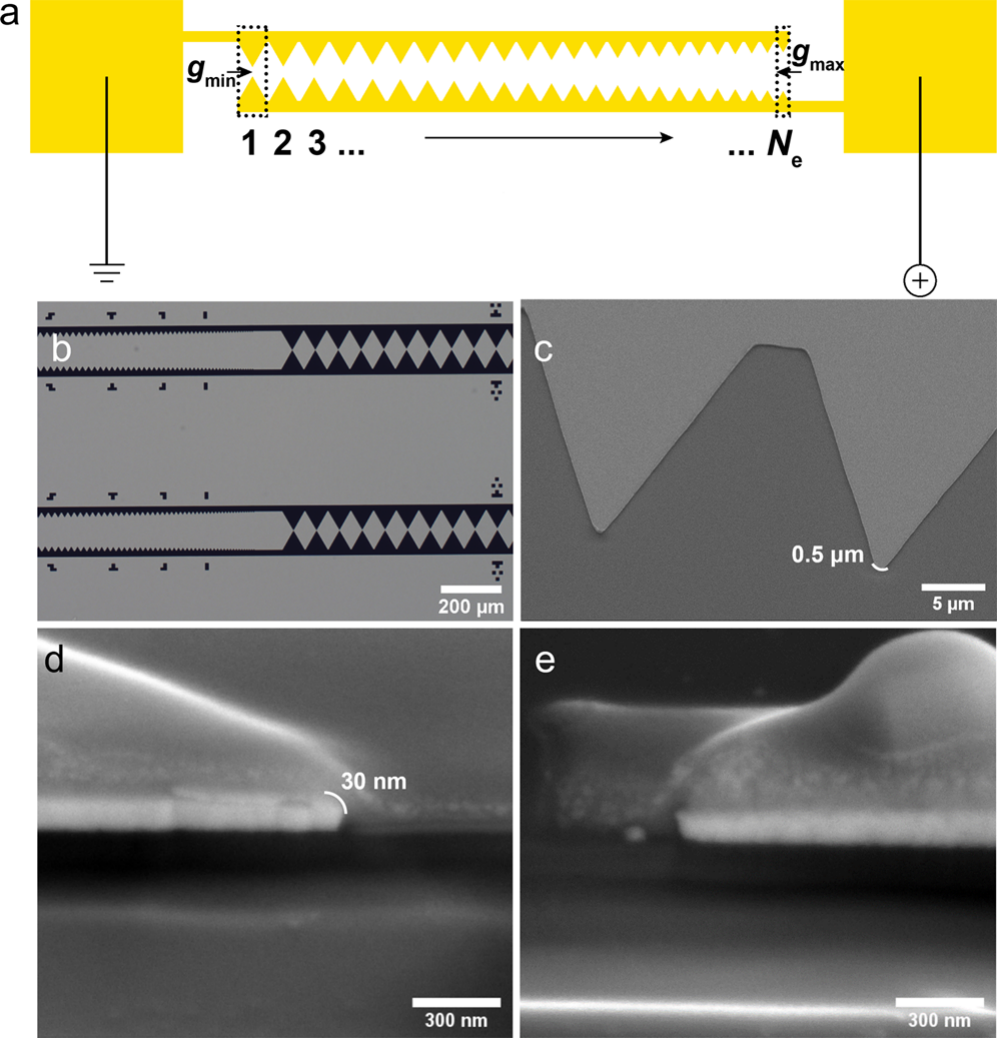
(a) Device design and corresponding (b) optical microscope and (c) SEM images (top views). (d, e) FIB SEM side view images used to measure the electrode apex radius of curvature.

The design of the dielectrophoretic device and the corresponding scanning electron microscopy (SEM) images are shown in Figure 2. The design consists of two parallel lines connected to square contact pads (Figure 2a). Each line represents an array of sawtooth electrodes with different sizes, building electrode pairs with varying *g*. In total, four pairs of lines are connected to one pair of contact pads, while the whole device contains four such ensembles. Two different designs with various numbers of electrode pairs, *N*_e_, were developed in this work. The first one includes *N*_e_ = 215 sawtooth tips separated by gaps varying between 2 μm (*g*_min_) and 130.5 μm (*g*_max_). The corresponding gap distance increment Δ*g* between two adjacent tip pairs is about 0.6 μm. The second design includes *N*_e_ = 90 sawtooth tips with *g*_min_ = 30 μm, *g*_max_ = 375.7 μm, and Δ*g* about 3.8 μm. Both devices were connected to a function generator using macroelectrodes by making a mechanical contact with the pads. The optical and field-emission SEM images for the fabricated devices are shown in Figure 2b,c, while focus ion beam SEM images are represented in Figure 2d,e. These images demonstrate the achieved fabrication reproducibility for the electrode arrays and show the electrode apex radius of curvature, one important geometrical parameter for the following electromagnetic simulations.

### Numerical Simulation

**Figure 3:**
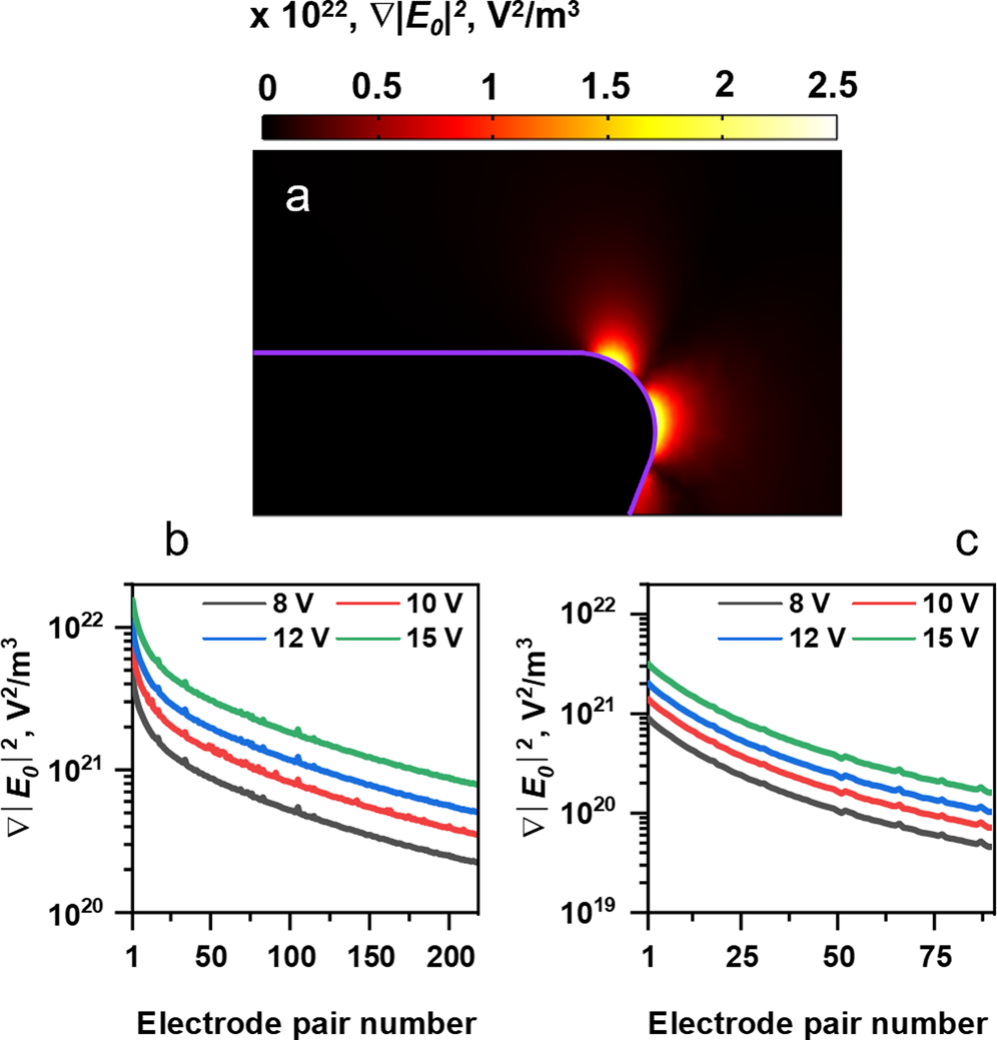
(a) Electric field gradient intensity distribution computed after applying 10 V for a three-dimensional model representing the cross-sectional view of the fabricated sawtooth electrodes. Electric field gradient intensity as a function of the electrode pair for different applied voltages and the designs with (b) *N*_e_ = 215 electrodes or (c) *N*_e_ = 90 electrodes.

The results of electromagnetic FEM 3D simulations are shown in Figure 3. They were performed using the AC/DC module of COMSOL Multiphysics 5.6. A 3D model representing the cross-sectional view of the fabricated sawtooth electrodes for two different device designs was constructed to estimate the magnitude of |*E*_0_| and ∇|*E*_0_|^2^ (Figure 3a). This model is based on the effectively fabricated geometry, as shown in Figure 2d,e. Figures 3b,c, show for different applied voltages, the calculated ∇|*E*_0_|^2^ values for each electrode pair within the device with *N*_e_ = 215 and *N*_e_ = 90, respectively. It is important to note that throughout this work the magnitudes for |*E*_0_| and ∇|*E*_0_|^2^ are obtained near the electrode corner observed from a cross-sectional view of the structure (Figures 2d and 3a). Indeed, since we perform 3D simulations, we could have used instead a top view of the structure at a vertical position equal to the electrode thickness (see the Supporting Information); however, in this case, the magnitude of the electric field intensity gradient is about 2.5 times weaker This difference can be explained by the different radii of curvature observed at the end of the electrode tip, namely, 50 nm for the cross-sectional view (Figures 2d and 3a) and 500 nm for the top view (Figures 2c and S2): the shortest radius of curvature producing the strongest field gradient.^51^ Considering the proportionality between the DEP force and the intensity of the EF gradient (eq 2), it is thus obvious that the protein molecules are accumulated and therefore observed near the region of shortest curvature. Hence, we applied the maximum intensity magnitude of EF gradient simulated for the cross-sectional view for the calculations of CM_micro_ by eq 3.

We also performed a nonlinear fit for the curves depicted in Figure 3b,c and found an exponential dependence of the electric field gradient on the electrode spacing (viz. the electrode pair number). These results are consistent with previous findings^52,53^ and reflect the importance of an electrode geometry choice for controlling the DEP force strength within the device.

### DEP of Proteins

**Figure 4:**
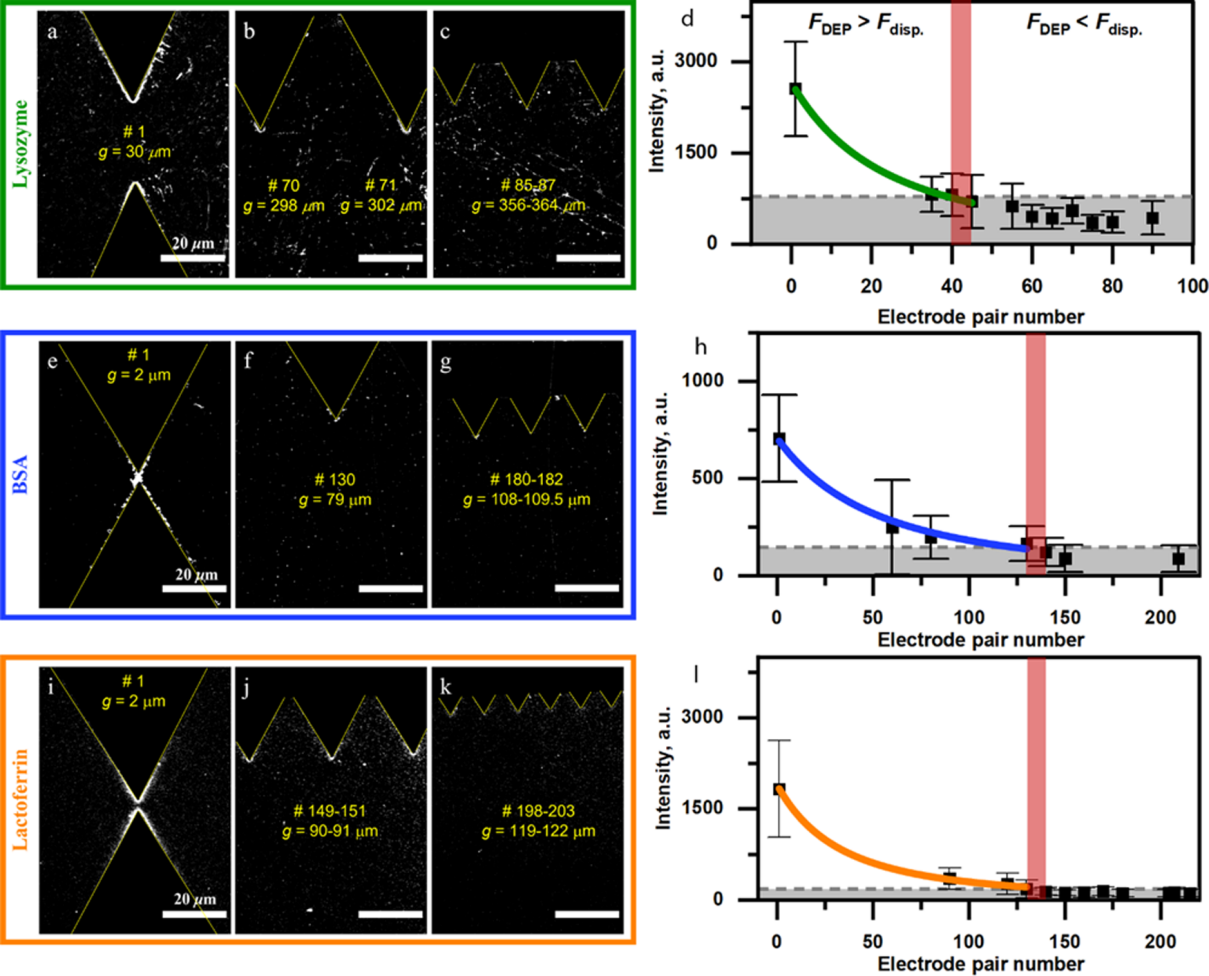
Confocal fluorescent images acquired for lysozyme (a–c), BSA (e–g), and lactoferrin (i–k) after DEP at 15 V and 0.3 MHz. The images were acquired for electrode pairs #1 (a), #70–71 (b), and #85–87 (c) of the device with *N*_e_ = 90 and for electrode pairs #1 (e, i), #130 (f), #180–182 (g) #149–151 (j), and #198–203 (k) of the device with *N*_e_ = 215. The electrode pair number and the corresponding gap *g* are indicated in yellow; all scalebars are 20 μm. The quantitative analysis of the fluorescence intensity originating from different electrode pairs after 10 min of DEP of lysozyme (d), BSA (h), and lactoferrin (l) conducted at 10 V and 0.3 MHz.

Figure 4 shows the confocal fluorescence images acquired for three different proteins labeled with FITC after DEP for 10 min at an applied voltage of 10 *V*_pp_ and a frequency of 0.3 MHz. The yellow dashed lines represent the contour of the metal electrodes, and the electrode pair number is indicated by yellow labels. The proteins are dissolved in 5 mM HEPES buffer (pH = 7.4, conductivity 167.5 μS/cm) with a concentration of 500 ng/mL. Lysozyme is studied in Figure 4a–c, BSA is investigated in Figure 4e–g, and lactoferrin is studied in Figure 4i–k. The *N*_e_ = 90 device is used for lysozyme, while that with *N*_e_ = 215 is used for BSA and lactoferrin. Similar fluorescence images for other applied voltages and electrode pairs can be found in the Supporting Information (Figures S5–S7).

Upon the application of the external AC signal, the accumulation of proteins begins in the gap of the first electrode pair and rapidly progresses to the neighboring pairs (see the Supporting Information Movie S1 for the FITC-labeled lysozyme), indicating biomolecular movement toward electrode edges (positive DEP) induced by various DEP forces generated in the EF gradient (see eq 2). It is also seen from Figure 4 that the increase in the electrode gap leads to a fluorescence intensity drop near the electrode edges (region of interest; Figure S6). When the gap reaches a specific value, no fluorescence is observed for the electrode pairs with larger gaps, albeit a weak fluorescence background from the molecules randomly adsorbed across the surface of the device. This fluorescence threshold indicates the transition between trapping and nontrapping regions, where the DEP or dispersion force is dominant, and can be correlated with the EF intensity gradient obtained from the corresponding numerical simulations. Since each protein has a different polarizability, one needs to select the correct device, such that this transition between trapping and nontrapping regions is well visible. Here, we use the device with *N*_e_ = 215 for BSA and lactoferrin. For lysosome, no fluorescence threshold was observed with that device (meaning that all electrode pairs produce a strong enough EF intensity gradient to trap lysosome) and we had to resort to the *N*_e_ = 90 device, which has larger gaps and produces weaker EF intensity gradients to clearly identify the fluorescence threshold for that protein.

### DEP Result Analysis and Model Validation

Figure 4 also shows the quantitative analysis of the fluorescence intensity as a function of the different electrode pair numbers for lysozyme (Figure 4d), BSA (Figure 4h), and lactoferrin (Figure 4l). Square dots represent the mean fluorescence intensity calculated within the region of interest (see the Supporting Information, Figure S8), while its standard deviation determines the vertical bars. The gray regions in Figure 4 indicate the regions below the 95th percentile of the background fluorescence distribution, which define a limit where fluorescence is not anymore distinguishable from the background. The fluorescence intensity values above that limit were considered significant in comparison to the background and used for the nonlinear fitting. This fitting (*R*^2^ ∼0.97) has the same exponential dependence on the electrode pair number as the EF intensity gradient (Figure 3b,c). For those values, the observed intensity is due to DEP trapping of proteins rather than random adsorption onto the surface. The crossover region indicated by the red vertical band represents the fluorescence intensity threshold. It defines the transition between DEP forces stronger (on the left) and weaker (on the right) than the dispersion forces acting on a protein. Using the calculated ∇|*E*_0_|^2^ value (Figure 3b,c) for the electrode pair corresponding to the left of the crossover region, one obtains the minimum ∇|*E*_0_|^2^ value required to trap the protein. This value can then be used in eq 3 to calculate CM_micro_ for that protein. The results of these calculations, as well as values reported by Hölzel and Pethig, are presented in Table 1.^26,27^ For clarity, this table also includes a column with the minimum ∇|*E*_0_|^2^ values required for proteins to overcome the dispersive force, which are predicted by the standard macroscopic DEP theory, assuming CM_macro_ = 1. Besides, the values indicated in parentheses in columns 2 and 4 in Table 1 are obtained by considering the hydrodynamic radius of proteins measured with the dynamic light scattering technique^54^ (see the Supporting Information for more details).

It is seen from Table 1 that the experimentally obtained CM_micro_ values are different from those derived using the dielectric spectroscopy by the empirical model in eq 1. For lysozyme, the experimental value is about 3.5 times lower (402 ± 50 against 1′390), while it is about 2 orders of magnitude lower for BSA (5 ± 0.65 against 1′110). No dielectric spectroscopy data exist for lactoferrin, while our experiments provide a value CM_micro_ = 3 ± 0.5, which is close to that obtained for BSA.

### Experimental Error on the Observed CM_micro_ Values

Let us discuss the effect of possible sources for the observed discrepancy between the experimental CM_micro_ values and those reported in the literature. The first most obvious source of experimental error may be related to the fluorescence intensity measurements. These measurements directly determine the threshold ∇|*E*_0_|^2^ values that are used for CM_micro_ calculations via eq 3. In this work, we followed the limit of the blank defined in the norm ISO 11,843 to define the significant fluorescence intensity threshold.^55^ This means that we utilized the 95th percentile of the background fluorescence distribution as a limit for distinguishing significant fluorescence intensity values. Hence, when the mean fluorescence intensity distribution measured at a given electrode pair is equal to this limit of the blank, then 50% of the measured fluorescence distribution in that region is significant, i.e., statistically different from the background fluorescence.^55−57^ This limit is high enough for our purpose because of the following reasons. Since the mean intensities and corresponding standard deviations depicted in Figure 4d,h,l are determined by postprocessing of digital fluorescence images (Figure 4a–c,e–g,i–k), analyzing the same fixed size image region (Figure S6), the identical number of pixels is always involved in the calculation. However, the protein distribution near the electrodes changes upon increasing the gap size: accumulation and therefore fluorescence emission occur mainly near the electrode tips and drops rapidly along the sides of the electrodes. Since our chosen region of interest (Figure S6), where the fluorescence emission is calculated, encompasses not only the electrode apex but also part of the triangular electrode sides, the total measured mean fluorescence intensity and standard deviation values for the electrodes with larger gaps are thus smeared out by the image pixels without fluorescence along these sides. As a result, the measured mean intensity value becomes closer to the background intensity, while the electrode apex still exhibits distinguishable fluorescence. Therefore, these electrodes cannot be ignored, even if about half of the fluorescence intensity is below the established threshold value.

We could have used as a significant fluorescence intensity threshold the limit of detection defined in ISO 11,843,^55^ which is placed two standard deviations higher than the limit of the blank and is used, e.g., to eliminate false-positive results in clinical trials.^57^ In that case, the gray regions in Figure 4d,h,l become higher, and the threshold regions (red bands in the same figures) move to the left, i.e., toward smaller electrode gaps. Subsequently, the calculated CM_micro_ becomes smaller for all proteins. This produces a larger statistical difference between the background and fluorescence of interest but increases the discrepancy between the experimental CM_micro_ values and those reported in the literature for lysozyme and BSA. We can argue that if the observed discrepancy was caused by an inappropriate choice of the fluorescence intensity threshold, then the reinforcement of the threshold should bring the experimentally obtained CM_micro_ values closer to the reported ones, which is not the case here.

Next, we examine the possible influence of the ∇|*E*_0_|^2^ simulation accuracy. In this work, the finite-element method was utilized for the simulations. This means that the values of calculated spatial derivatives for the potential distribution (i.e., ∇|*E*_0_|^2^ and |*E*_0_|) strongly depend on the defined model geometry and the chosen mesh resolution. Correct EF simulations require an accurate knowledge of the electrode geometry, especially its corners. We have taken great care to define the geometry from FIB SEM images (Figure 2d,e) and optimized the mesh resolution such that the results for ∇|*E*_0_|^2^ are converged to a nearly constant value within a reasonable computation time (the chosen mesh element size is 5 nm; see the Supporting Information, Figure S3). Of course, the actual radius of curvature may vary between different electrode pairs due to fabrication imperfections. Moreover, applied voltage instabilities may also play a role in the real ∇|*E*_0_|^2^ values established during experiments. However, repeated measurements confirmed the reproducibility of the determined DEP threshold, and we can assume that the obtained CM_micro_ values are weakly dependent on this source of error.

Although it cannot be completely excluded, it is very unlikely that the interaction between DEP and electrothermal effects could have played a role here. Indeed, relatively high-frequency (300 kHz) and low-conductivity buffers (σ_m_ = 167.5 μS/cm) were utilized in the experiments.^42−45^ The same low-conductivity conditions as well as low protein concentrations (500 ng/mL) and physiological pH of the utilized buffer (7.4) prevent changes of the protein molecular properties (size, for example) caused by aggregation and thus ensure that DEP is done on a molecular scale rather than the macroscale (see the Supporting Information for additional details).^26,58−60^

Another important factor to consider is the proper definition of the protein volume in eq 3. We know so far that the majority of proteins are ellipsoids rather than spheres, and in water they form a conglomerate with neighboring water molecules, which acts as a single entity during DEP. Therefore, the simple incorporation of a sphere volume in eqs 2 and 3, which is equal to the boundary matching physical dimension of the sole protein molecule, is strictly speaking not correct, and different approximations should be considered. This can be done, for example, through the recently proposed modification of the model in eq 3, which replaces the sphere volume with the so-called Fröhlich volume *V*_Fr_ of the protein in the water medium.^27,61^ Within this volume, the water molecules of protein hydration interact with the protein structure and its dipole field to such an extent that their effective permittivity does not match the medium permittivity value given in eq 3. The radius of the Fröhlich volume thus replaces the radius *R* of the protein molecule in eq 3. Its value can be calculated for lysozyme and BSA based on the experimental dipole moment μ. Assuming static dielectric constant ε_p_ = 25 for both proteins and μ = 122 D for lysozyme and μ = 710 D for BSA, this gives *V*_Fr_ = 73 nm^3^ for lysozyme and *V*_Fr_ = 2474 nm^3^ for BSA. The corresponding CM_micro_ values are reported in Table 1 for both proteins; they disagree more with the values reported in the literature while bringing them closer to the classical CM_macro_ definition. For BSA, the value even falls within the range defined by the classical DEP theory (−0.5 < CM_macro_ < 1).

Overall, we have not been able to experimentally reproduce the CM_micro_ values published in the literature. We have measured an approx. 3.5 times lower CM_micro_ value for lysozyme and an approx. 2 orders of magnitude lower value for BSA (Table 1). We also obtained CM_micro_ for lactoferrin, for which no dielectric spectroscopy data exist. In any case, the possible shortcomings of classical theories for describing DEP of proteins observed here are consistent with the observations that have prompted the development of more sophisticated theories^26,27,32,33^ and previous experimental findings,^14−20,28^ indicating that the standard macroscopic DEP theory fails to describe protein DEP. The protein DEP response is usually observed upon generating much lower EF intensity gradients compared to those required to overcome dispersive forces.

## Conclusions

In summary, we have experimentally studied DEP for three model proteins, namely, lysozyme, BSA, and lactoferrin, using a device built from an array of metal electrode pairs with varying gaps, which produces variations of the EF gradient intensity. This device can effectively determine the transition between two competing forces: DEP and the dispersive force associated with Brownian motion. This transition has been determined by the last electrode pair with observable fluorescence from the trapped proteins. The DEP force and molecular CM_micro_ function have been calculated according to eq 3 by using the numerically simulated intensity of EF gradient for the threshold electrode pair. We have compared the obtained CM_micro_ values with those calculated by the recently reported empirical model of eq 1 and found a discrepancy of about 3.5 times lower for lysozyme and 2 orders of magnitude lower for BSA. The possible sources of experimental error have been critically evaluated, and we concluded that they should have only a very minor influence.

The presented experimental findings are generally consistent with the known failure of the classical DEP theory when applied to protein DEP. We have also shown that even with incorporation of the most recent developments of the protein DEP theory, the experimental results are still far from the calculations.

Let us conclude by noting that the experimental technique established here to determine the minimum DEP trapping threshold and the corresponding molecular CM_micro_ function may be utilized for any protein molecule, thus providing an efficient approach to expanding experimental DEP data and refining protein DEP theory. These advances will set the ground for developing new types of biosensors able to manipulate, sort, and trap proteins at the microscale.

## Supporting Information Available

Details on the influence of protein aggregation, solvent properties, and electrothermal and AC electroosmotic fluid flow on CM_micro_ values; experimental details for the DLS measurements; figures showing the DLS measurement results for protein solutions (Figure S1); simulated electrothermal fluid flow velocities (Figure S2); simulated intensity of the EF gradient dependency on mesh element size (Figure S3); simulated intensities of electric field gradient distribution for the top view of the three-dimensional electrode model (Figure S4); confocal fluorescent images acquired after 10 min of DEP at 8, 10, and 12 V for lysozyme (Figure S5); BSA (Figure S6); lactoferrin (Figure S7); and region of interest for fluorescence intensity calculations (Figure S8) (PDF)
DEP accumulation of proteins near sawtooth electrode edges (Movie S1) (AVI)
