# Supplementary material for: Protein Dielectrophoresis with Gradient Array of Conductive Electrodes Sheds New Light on Empirical Theory
Source: Anal Chem. 2023 Jan 24;95(5):2958–66. doi: 10.1021/acs.analchem.2c04708 (PMC9909730; doi:10.1021/acs.analchem.2c04708)
Supplement: Supplementary file 1 — ac2c04708_si_001.pdf [file ac2c04708_si_001.pdf]

Supporting Information

Protein Dielectrophoresis with Gradient Array of Conductive Electrodes Sheds New Light on Empirical Theory

Siarhei Zavatski[a*], Hanna Bandarenka[b], Olivier J.F. Martin[a*]

[a]Nanophotonics and Metrology Laboratory (NAM), Swiss Federal Institute of Technology Lausanne (EPFL), Lausanne 1015, Switzerland

[b]The Polytechnic School, Arizona State University, Mesa, Arizona 85212, United States

Corresponding authors

*E-mail: siarhei.zavatski@epfl.ch (S. Zavatski); olivier.martin@epfl.ch (Prof. O. J.F. Martin).

Table of contents

Influence of protein aggregation on the obtained CM_{micro} values…………………………………..…S2

Influence of solvent properties on the obtained CM_{micro} values……….………….……….……..…S5

Influence of electrothermal and AC electroosmotic fluid flow on the obtained CM_{micro} values….S7

Dynamic light scattering measurements for protein solutions...……………………………..….....S8

Figure S1. DLS measurements for protein solutions at concentration of 7 mg/mL………........…..S9

Figure S2. Simulated velocities of electrothermal fluid flow for 3D model representing the cross-section view of the fabricated sawtooth electrodes……………………………………………....S9

Figure S3. Intensity of electric field (EF) gradient dependency on mesh element size................S10

Figure S4. Simulated EF gradient intensity distribution for two-dimensional model………....S10

Figure S5. Confocal fluorescent images for lysozyme after DEP at different applied voltage….S11

Figure S6. Confocal fluorescent images for BSA after DEP at different applied voltage...........S12

Figure S7. Confocal fluorescent images for lactoferrin after DEP at different applied voltage..S13

Figure S8. Region of interest (ROI) for fluorescence intensity calculations…………………....S14

References…………………………………………………………………………..…..S15

Influence of protein aggregation on the obtained CM_{micro} values.

To confirm that protein agglomerates have a very low concentration compared to protein monomers and thus have minor influence on the results reported in our study, we have performed dynamic light scattering (DLS) measurements (Figure S1). These experiments also confirm the absence of other protein forms (e.g. dimers, trimers and others), which would exhibit a different dielectrophoretic (DEP) response compared to protein monomers but produce the same smooth fluorescent pattern due to the limited resolution of a microscope. Figure S1 indicates that the DLS intensity for BSA is almost completely within the band corresponding to protein monomers, providing hydrodynamic radius of 4.36 ± 1.77 nm, which is consistent with other studies.[1] However, DLS measurements for lysozyme, respectively lactoferrin, solutions revealed a second band at approx. 95 nm, respectively 30 nm, radius. Nevertheless, the scattered intensity for the monomer band of lysozyme (measured hydrodynamic radius of 1.81 ± 0.32 nm, which is also in agreement with previous studies (Ref. 33 in the main text)) and lactoferrin (hydrodynamic radius of 6.9 ± 1.81 nm; slightly larger compared to human lactoferrin,[2] yet consistent with more recent DLS studies on bovine lactoferrin[3,4]) is still about twice larger compared to the second band. However, the appearance of second bands does not indicate that the lysozyme and lactoferrin solutions are dominated by aggregates because the measured DLS intensity is proportional to R^6 (R is the hydrodynamic radius of a protein); in other words, even a minute content of large particulates can produce a significant DLS response. To estimate the volume concentration of these aggregates, we followed the procedure outlined elswhere,[5] which requires the utilization of the so-called correlation function produced during DLS measurements. For solutions with a total concentration of 7 mg/mL, estimation protocol yielded aggregate concentrations of $\sim 1 \cdot 10^7$ cm^{-3} for lysozyme and $\sim 9 \cdot 10^{13}$ cm^{-3} for lactoferrin. This represents very low amounts (specifically, $\sim 10^{10}$ and 500 times lower than the concentration of lysozyme and lactoferrin monomers), despite of the very high concentration used in those measurements. Indeed, we should note that DLS measurements require highly concentrated protein solutions (7 mg/mL), whilst the DEP experiments used considerably more diluted samples (500 ng/mL). Therefore, we can assume that the concentration of lysozyme aggregates in the DEP experiments is even lower. Specifically, assuming no changes in the protein solutions upon dilution, the concentration of lysozyme and lactoferrin aggregates in the DEP experiments would be 690 cm^{-3} and $6.5 \cdot 10^9$ cm^{-3}, respectively.

To further confirm the low concentration of aggregates in the solutions studied in this work, we estimated their number in the largest possible volume of the so-called depletion zone generated by the electrodes. This volume defines the region where the DEP force acting on a protein is larger than the corresponding dispersive forces. The aggregates located in this volume hence are assumed to be trapped by the electrodes. Considering that they behave as macroscopic particulates (i.e., classical DEP theory applies) with $CM_{macro} = 1$, our calculations give the maximum volume of the corresponding depletion zone as $2 \cdot 10^{-11}$ cm^3. For solutions concentrations of 500 ng/mL, this volume should encompass approx. $1.3 \cdot 10^{-8}$ and 0.129 aggregates of lysozyme and lactoferrin, respectively – clearly not enough to become a dominant source for the fluorescence intensity visible withing a few seconds upon electric field application (supplementary movie S1). Such aggregates could however be trapped and/or adsorb on the surface during longer 10 min DEP experiments, as the depletion zone is constantly replenished by the molecules approached from the bulk medium. Therefore, considering the kinetics of the fluorescence intensity generation reported in the supplementary movie S1 and the DLS results (Figure S1) confirming the presence of not more than two protein sizes, we can conclude that the protein DEP reported in this study is due to the trapping of protein monomers.

Influence of solvent properties (ionic strength and pH) on the obtained CM_{micro} values.

Before discussing this issue, let us recall that the calculations of CM_{micro} require the experimental dielectric spectroscopy data (see Eq. (1) in the main text). Specifically, the measurement of the dielectric increment $\Delta\varepsilon$, responsible for the β-dispersion of the protein (dipole orientation process). Therefore, it is useful to inspect the possible consequences of the difference between our experimental conditions and those applied for $\Delta\varepsilon$ measurements.

First, although most of dielectric spectroscopy studies have been accomplished in low conductivity media to increase measurement accuracy by preventing the polarization of electrodes, several studies have indicated a minor effect of the ionic strength on dielectric β-dispersion (i.e., $\Delta\varepsilon$ or protein dipole moment).[6–8] Moreover, considering the ionic strength used in the present study (5mM of HEPES), which implies about 10 HEPES molecules per protein, we can state with confidence that the ionic atmosphere of the proteins investigated here is fairly low, corresponding to those used for $\Delta\varepsilon$ measurements. Besides, Lumry and Yue have experimentally demonstrated that the use of zwitterions (like those that we have utilized in our work, 5 mM of HEPES) up to 0.224 M have not provided any prominent changes in the dielectric behavior of proteins.[9] As such, the discrepancy between experimental and calculated (by using $\Delta\varepsilon$ values) CM_{micro} is unlikely to be related with this source of error.

Second, previous investigations have indeed demonstrated that the dielectric increment $\Delta\varepsilon$ (dipole moment) of various proteins depends on the solvent pH.[6–8,10,11] In general, in the 4.5 – 10 solvent pH range, the theory predicts the monotonic increase of the protein dipole moment,[11] while the prediction outside of this range is scarce. This theoretical prediction has been confirmed by several experiments.[7,8,10,11] Generally, the variation of the dipole moment occurs due to the proton fluctuation near the protein, producing a considerable shift of the dipole moment magnitude.

In that regard, CM_{micro} should also depend on the solvent pH because it is directly proportional to $\Delta\varepsilon$, according to the new Hölzel & Pethig empirical theory (see Eq. (1) in the main text). To see if this dependency could lead to the discrepancy revealed in this work, let us first recall pH of the buffers utilized elsewhere to obtain $\Delta\varepsilon$ and thus to calculate CM_{micro} for BSA and lysozyme (see the last column of Table 1 in the main text). The buffer pH for BSA and lysozyme dielectric spectroscopy studies were 5.15 and 11.17 (isoelectric point),[12] respectively, which is different from that utilized in the present work (pH = 7.4). According to the above-mentioned theoretical model of protein dipole moment dependence on a solvent pH,

our experimental conditions hence should produce larger CM_{micro} value for BSA compared to the reported by Hölzel & Pethig, which is not observed in the present study.

On the other hand, we cannot perform the same analysis for lysozyme with $\Delta\varepsilon$ measured at a pH of 11.17 because it exceeds the above-mentioned range, where the values of the protein dipole moment can be adequately predicted theoretically. Nevertheless, more recent dielectric spectroscopy study for lysozyme performed at the buffer pH of 5.5 revealed a comparable $\Delta\varepsilon$ values. Hence, relying on this value and confirmed validity of the protein dipole moment monotonic increase in the 4.5–10 range of buffer pH,[7,8,10,11] we should also obtain the larger CM_{micro} value for lysozyme compared to the reported by Hölzel & Pethig, which is not the case here. Hence, we can assume that the CM_{micro} values reported here are weakly dependent on this source of error.

Influence of electrothermal and AC electroosmotic fluid flow on the obtained CM_{micro} values.

Several seminal studies (see e.g. Refs. 40–43 in the main text) have shown the importance of the electrothermal effect for DEP experiments. Sometimes, the electrothermally generated fluid velocity may be so large that the study of a DEP response from targeted particulates becomes very complicated (see Ref. 41 in the main text). Nevertheless, these comprehensive studies suggest that the electrothermal fluid velocity can be efficiently controlled by various experimental parameters, including electric field strength and frequency, temperature gradient, electric conductivity and permittivity of the solvent, providing a way to reduce its magnitude. They also indicate that the experimental determination of this flow is not trivial, especially in the case of proteins, because of their very small size. We have thus addressed this issue from a theoretical point of view, utilizing numerical (COMSOL) calculations. Figure S2 depicts the velocity of the electrothermal fluid flow near sawtooth electrode apexes immersed in a fluid with conductivity 0.01675 S/m (corresponding to the experimental condition, Fig. S2 a) or 1 S/m (corresponding to a much higher fluid conductivity, Fig. S2 b). The applied electric field corresponds to the peak-to-peak voltage amplitude of 10 V and the frequency of 300 kHz for both figures. This figure shows that the velocity for the low conductivity fluid is about 3 orders of magnitude lower (maximum about 150 μm/s) compared to the high conductivity case (maximum about 13 mm/s). This confirms our statement in the main text that a relatively high frequency (300 kHz) and low conductivity buffer (σ_m = 167.5 μS/cm) reduce the electrothermal effects.

Nevertheless, the fluid velocity magnitude alone cannot justify the absence of its influence on particles DEP. For this, one should compare both the particles and fluid velocities. The protein velocity, v_{DEP}, induced by the DEP force can be roughly estimated using values in Table 1 in the main text and Stokes' law: $v_{DEP} = F_{DEP}/(6\pi\eta R)$, where F_{DEP} is calculated by Eq. (2) in the main text, η is the viscosity of the suspending medium, and R is the protein hydrodynamic radius. Considering lysozyme and taking $CM_{micro} = 402$, $R = 2$ nm, $\nabla|E_0|^2 = 6.81\cdot10^{21}$ V^2/m^3 (which corresponds to the maximum EF gradient intensity for sawtooth pair #1 in the $N_e = 215$ design), this gives $F_{DEP} = 9.5\cdot10^{-11}$ N and $v_{DEP} = 2.5$ m/s, which is undoubtedly much larger than both low and high conductivity fluid velocities. Even upon considering the threshold electrode pair for lysozyme of $\nabla|E_0|^2 = 1.84\cdot10^{20}$ V^2/m^3, this gives $F_{DEP} = 2.6\cdot10^{-12}$ N and $v_{DEP} = 66$ mm/s, which is of the same order with the high conductivity fluid movement but still much higher than the low conductivity fluid utilized in our study.

To estimate the effect of AC electroosmosis in our experiments, we followed the procedure outlined by Castellanos et al. in 2003.[13] This produced a maximum AC electroosmosis fluid velocity of approx. 8.44 mm/s at the applied voltage of 10 $V_{p\text{-}p}$ and the frequency of ~67 kHz, while the buffer conductivity was 0.01675 S/m. The calculated frequency, where the electroosmotic fluid flow becomes maximum, is more than 4 times lower compared to that applied in this work (300 kHz). The maximum AC electroosmosis fluid velocity above the surface of our electrodes at the experimental frequency of 300 kHz is assumed to be around 21.5 μm/s, which is much lower compared to the predicted protein (lysozyme) velocity (2.5 m/s for the lowest electrode gap and 67 mm/s for the threshold pair of electrodes).

Dynamic light scattering measurements from protein solutions

Dynamic light scattering data were collected with an Zetasizer Nano ZS equipped with He-Ne laser (4 mW, 632.8 nm). Prior to loading in the cuvette, the protein solution with 7 mg/mL concentration was filtered through 0.1 μm Whatman Anotop syringe filters. All measurements were performed in quartz cuvette at room temperature and a measurement angle of 173°.

Supporting Figures

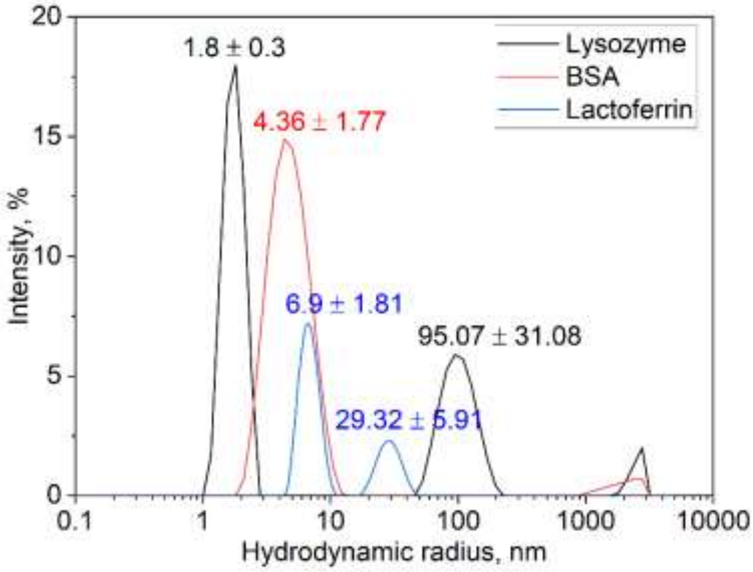

Figure S1. DLS measurements for protein solutions at a concentration of 7 mg/mL.

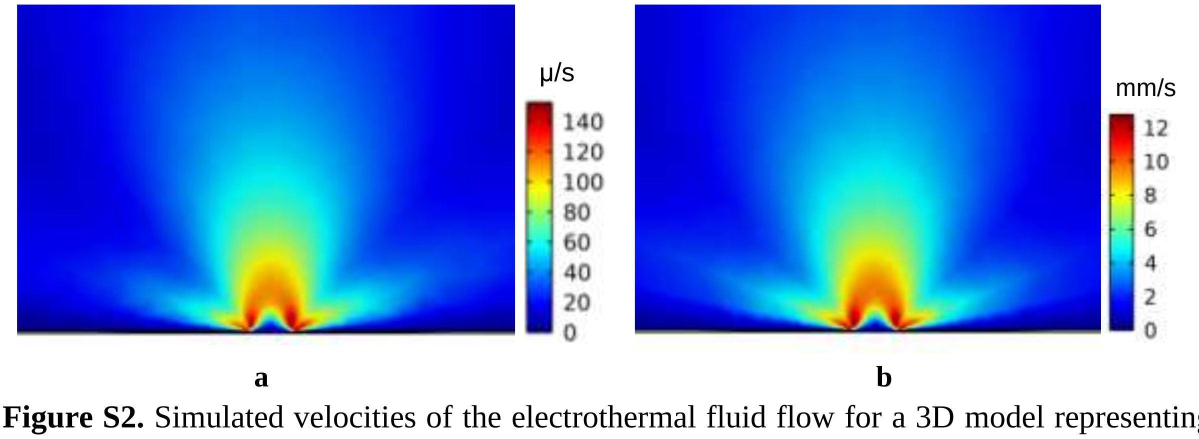

Figure S2. Simulated velocities of the electrothermal fluid flow for a 3D model representing the cross-section view of the fabricated sawtooth electrodes. Fluids with conductivity of (a) 167.5 μS/cm and (b) 1 S/m.

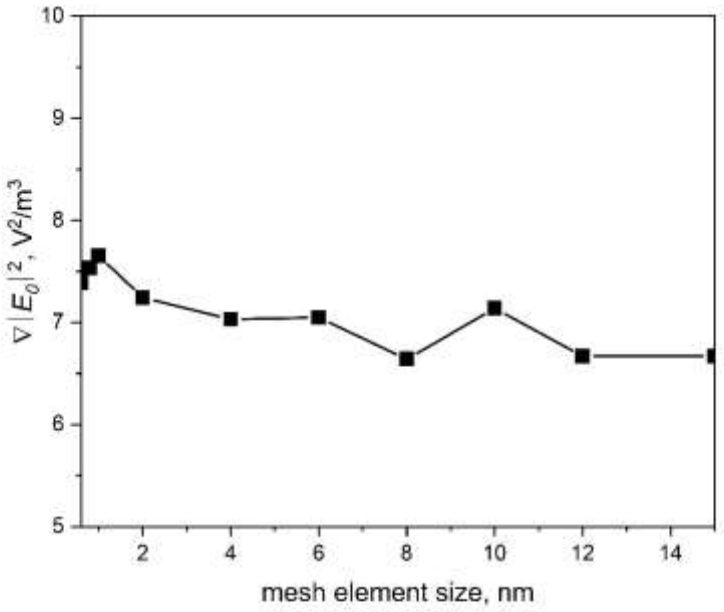

Figure S3. Intensity of electric field (EF) gradient dependency on the mesh size calculated for electrode pair with a 2 μm gap at an applied voltage of 10 V and a frequency of 0.3 MHz. The calculated EF gradient intensity is almost constant in the 0.6–15 nm range of the studied mesh element sizes. Similar results were obtained for other electrode pairs energized at different voltages (data not shown).

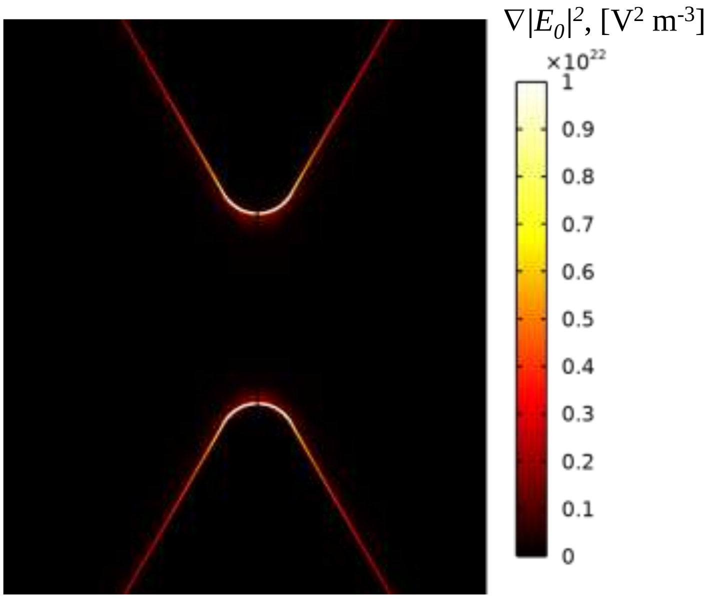

Figure S4. Simulated intensities of the electric field gradient distribution for a two-dimensional model representing the top view of the fabricated sawtooth electrodes.

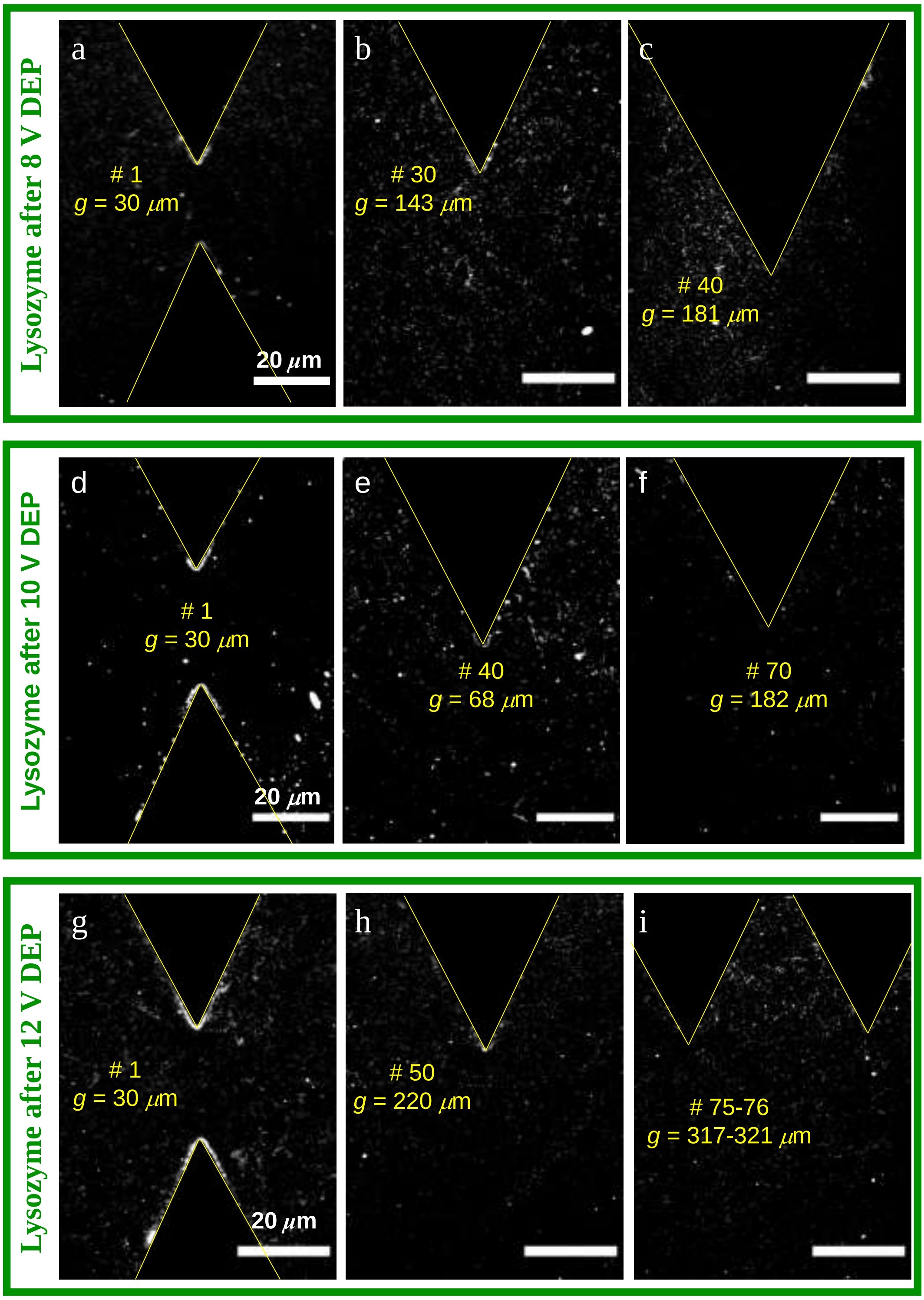

Figure S5. Confocal fluorescent images acquired after 10 min DEP at different applied voltages, indicated on the left of each row, for lysozyme near 1 (a, d, g), 30 (b), 40 (c, e), 70 (f), 50 (h), and 70–76 (i) electrode pairs of the device with $N_a = 90$.

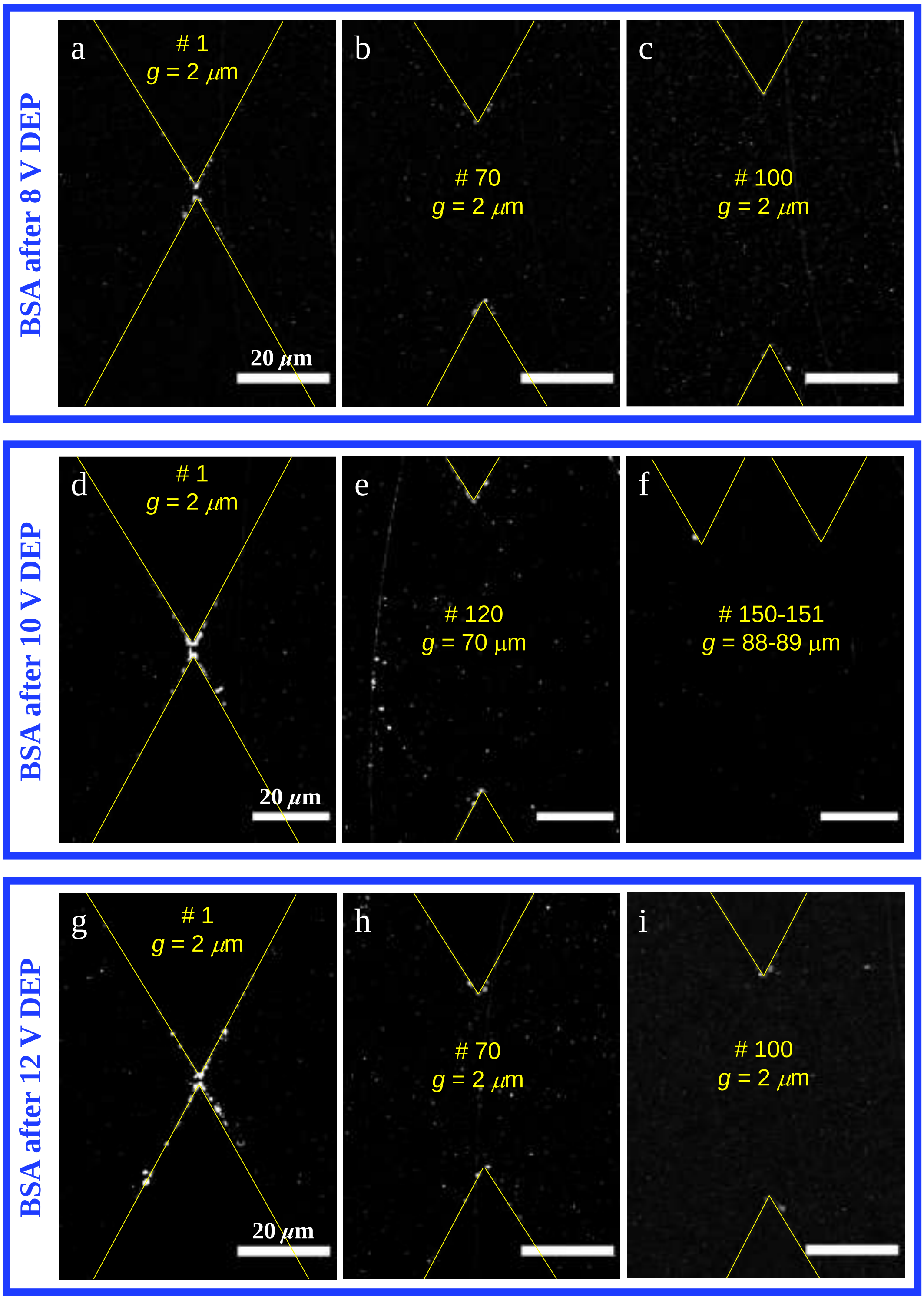

Figure S6. Confocal fluorescent images acquired after 10 min DEP at different applied voltages, indicated on the left of each row, for BSA near 1 (a, d, g), 70 (b, h), 100 (c, i), 120 (e), and 150–151 (f) electrode pairs of the device with N_a = 215.

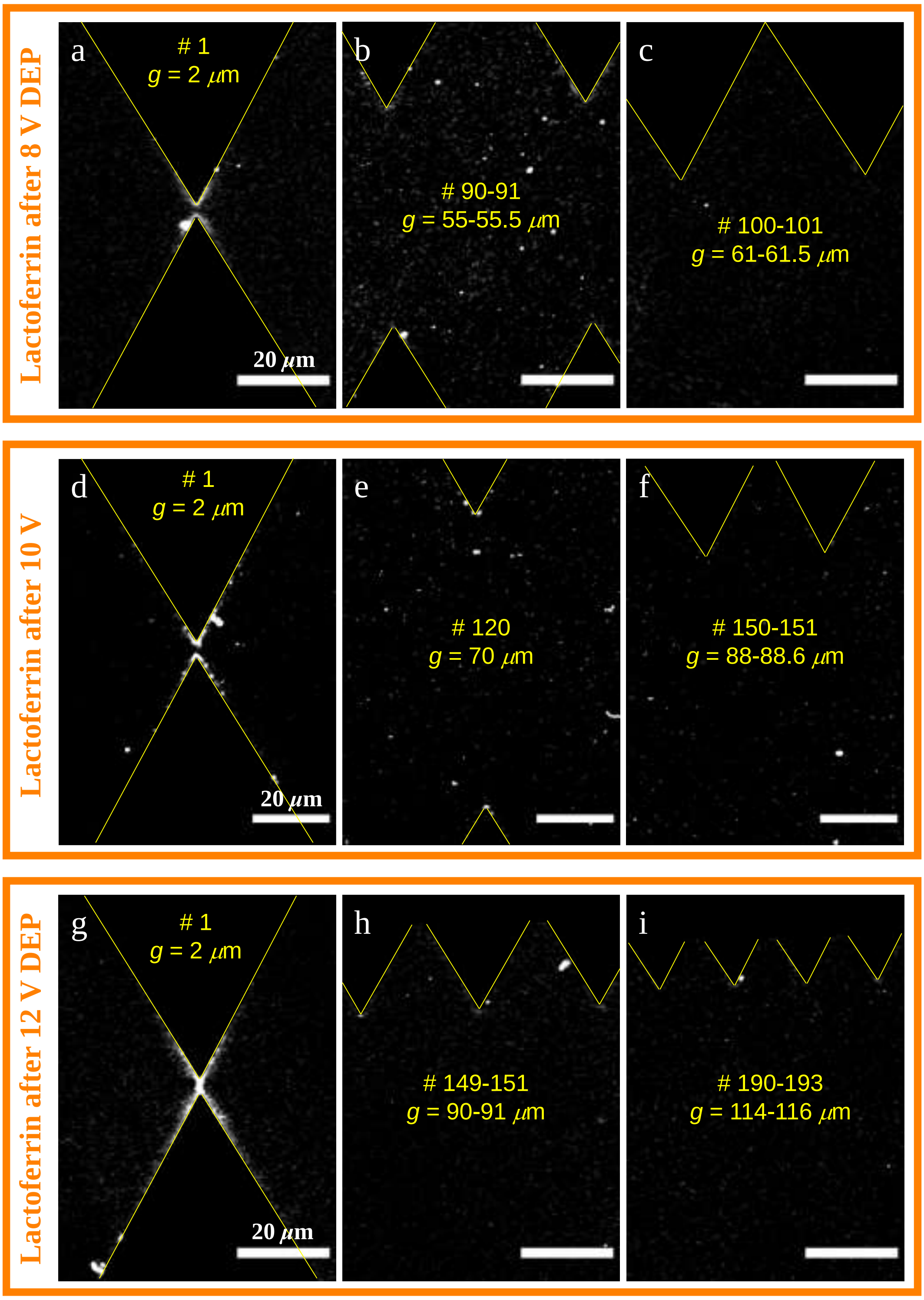

Figure S7. Confocal fluorescent images acquired after 10 min DEP at different applied voltages, indicated on the left of each row, for lactoferrin near #1 (a, d, g), #90–91 (b), #100–101 (c), 120 (e), 150–151 (f), and 149–151 (h), and 190–193 (i) electrode pairs of the device with N_a = 215.

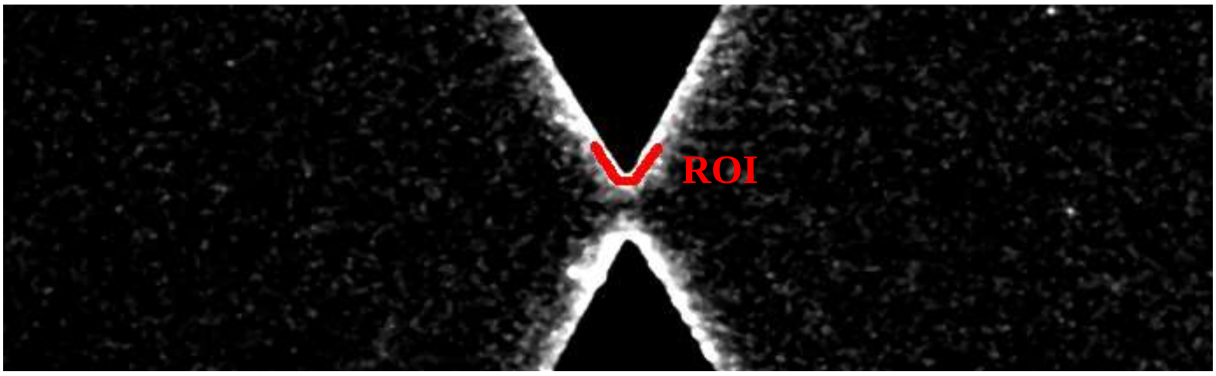

Figure S8. Region of interest (ROI) for the fluorescence intensity calculations.

References

(1) Valstar, A.; Almgren, M.; Brown, W.; Vasilescu, M. *Langmuir* **2000**, *16* (3), 922–927. https://doi.org/10.1021/la990423i.

(2) Tomitaka, A.; Arami, H.; Gandhi, S.; Krishnan, K. M. *Nanoscale* **2015**, *7* (40), 16890–16898. https://doi.org/10.1039/c5nr02831k.

(3) Saraiva, C. S.; dos Reis Coimbra, J. S.; de Carvalho Teixeira, A. V. N.; de Oliveira, E. B.; Teófílo, R. F.; da Costa, A. R.; de Almeida Alves Barbosa, É. *Food Res. Int.* **2017**, *100*, 674–681. https://doi.org/10.1016/j.foodres.2017.07.065.

(4) Adal, E.; Sadeghpour, A.; Connell, S.; Rappolt, M.; Ibanoglu, E.; Sarkar, A. *Biomacromolecules* **2017**, *18* (2), 625–635. https://doi.org/10.1021/acs.biomac.6b01857.

(5) Li, Y.; Lubchenko, V.; Vekilov, P. G. *American Institute of Physics AIP*, **2011**; Vol. 82. https://doi.org/10.1063/1.3592581.

(6) Mofers, F. J., Veltkamp, P. J., Van Faassen, E. E., Casteleijn, G., & Levine, Y. K. Biophysical Chemistry, **1982**, *16* (1), 19–25.

(7) South, G. P.; Grant, E. H. *Proc. R. Soc. London. A. Math. Phys. Sci.* **1972**, *328* (1574), 371–387. https://doi.org/10.1098/rspa.1972.0083.

(8) Bonincontro, A.; De Francesco, A.; Onori, G. *Colloids Surfaces B Biointerfaces* **1998**, *12* (1), 1–5. https://doi.org/10.1016/S0927-7765(98)00048-4.

(9) Lumry, R.; Yue, R. H. *J. Phys. Chem.* **1965**, *69*, 1162–1174. https://doi.org/10.1021/j100888a013.

(10) Mellor, B. L.; Cruz Cortés, E.; Busath, D. D.; Mazzeo, B. A. *J. Phys. Chem. B* **2011**, *115* (10), 2205–2213. https://doi.org/10.1021/jp1111873.

(11) Takashima, S. *J. Phys. Chem.* **1965**, *69* (7), 2281–2286. https://doi.org/10.1021/j100891a023.

(12) Kuehner, D. E.; Engmann, J.; Fergg, F.; Wernick, M.; Blanch, H. W.; Prausnitz, J. M. *J. Phys. Chem. B* **1999**, *103* (8), 1368–1374. https://doi.org/10.1021/jp983852i.

(13) Castellanos, A.; Ramos, A.; González, A.; Green, N. G.; Morgan, H. *J. Phys. D. Appl. Phys.* **2003**, *36* (20), 2584–2597. https://doi.org/10.1088/0022-3727/36/20/023.
